# Screening Collagenase Activity in Bacterial Lysate for Directed Enzyme Applications

**DOI:** 10.3390/ijms22168552

**Published:** 2021-08-09

**Authors:** Ran Tohar, Tamar Ansbacher, Inbal Sher, Livnat Afriat-Jurnou, Evgeny Weinberg, Maayan Gal

**Affiliations:** 1 Department of Oral Biology, The Goldschleger School of Dental Medicine, Sackler Faculty of Medicine, Tel Aviv University, Tel Aviv 6997801, Israel; tohar.ran@gmail.com (R.T.); tamar.ansbacher@mail.huji.ac.il (T.A.); inbalsher@mail.tau.ac.il (I.S.); evgenywein@gmail.com (E.W.); 2 Hadassah Academic College, 7 Hanevi’im Street, Jerusalem 9101001, Israel; 3 Migal-Galilee Research Institute, Kiryat Shmona 11016, Israel; livnatJ@migal.org.il; 4 Faculty of Sciences and Technology, Tel-Hai Academic College, Upper Galilee 1220800, Israel

**Keywords:** collagenase, enzymatic assay, molecular dynamics, protein expression, bacterial lysate screening, directed enzyme evolution

## Abstract

Collagenases are essential enzymes capable of digesting triple-helical collagen under physiological conditions. These enzymes play a key role in diverse physiological and pathophysiological processes. Collagenases are used for diverse biotechnological applications, and it is thus of major interest to identify new enzyme variants with improved characteristics such as expression yield, stability, or activity. The engineering of new enzyme variants often relies on either rational protein design or directed enzyme evolution. The latter includes screening of a large randomized or semirational genetic library, both of which require an assay that enables the identification of improved variants. Moreover, the assay should be tailored for microplates to allow the screening of hundreds or thousands of clones. Herein, we repurposed the previously reported fluorogenic assay using 3,4-dihydroxyphenylacetic acid for the quantitation of collagen, and applied it in the detection of bacterial collagenase activity in bacterial lysates. This enabled the screening of hundreds of *E. coli* colonies expressing an error-prone library of collagenase G from C. histolyticum, in 96-well deep-well plates, by measuring activity directly in lysates with collagen. As a proof-of-concept, a single variant exhibiting higher activity than the starting-point enzyme was expressed, purified, and characterized biochemically and computationally. This showed the feasibility of this method to support medium-high throughput screening based on direct evaluation of collagenase activity.

## 1. Introduction

Collagenases are essential components of the matrix metalloproteinases family of proteins [1]; their main function is the breaking of the triple-helix collagen. The latter is the major structural protein in many extracellular elements, including skin, bone, and dentin, and is the major connectivity protein within the extracellular matrix (ECM) in vertebrates [2]. Owing to its high abundance, the degradation of collagen and remodeling of the ECM are essential cellular processes. In addition to having important functions in vertebrates, collagenases also are found in various bacteria [3]. The bacterial enzymes are efficient in cleaving collagen at multiple sites, breaking it down to short peptide fragments [4]. Due to their ability to digest collagen in the ECM, bacterial collagenases are considered as important virulence factors, together with additional degrading enzymes such as elastase [5,6]. These enzymes assist in destroying extracellular structures, enabling efficient host colonization and penetration into anaerobic sites and promoting spread of infection. The realization of the important role of bacterial collagenases has promoted further research into the biochemical and structural properties of this family [4,7,8,9]. Moreover, bacterial collagenases have been explored as promising targets for inhibiting bacterial invasion without affecting viability of the bacteria [10,11,12,13]. Whereas the above calls for collagenase inhibition, many applications may benefit from optimized collagenase variants that harbor higher activity and stability. These include therapeutic applications, such as for Dupuytren’s and Peyronie’s diseases, and for treatment of burns and wounds [14,15,16,17,18,19,20]. Moreover, additional applications of collagenases are used in the agriculture and food industries, such as for controlling plant pathogens and for meat tenderness [21,22].

The range of research questions and applications that stem from various functional aspects of collagenases requires an efficient activity assay. Such a setup must be cost-effective, robust, and amenable to further adaptation to automated high throughput screening platforms. However, the majority of currently available assays for monitoring enzymatic activity of collagenases are based on a fluorescently labeled peptide mimicking a single collagen unit, or on the detection of hydroxyproline [23,24,25,26,27,28]. Antibodies have also been used to detect specific collagen fragments [29]. Additional methods consist of picrosirius red staining [30,31] or by labeling of newly form *N*-termini based on fluorescamine [32]. These techniques are relatively costly, may not rely on native collagen, may not fit high throughput screening, and often require harsh hydrolysis protocols for collagen.

**Figure 1:**
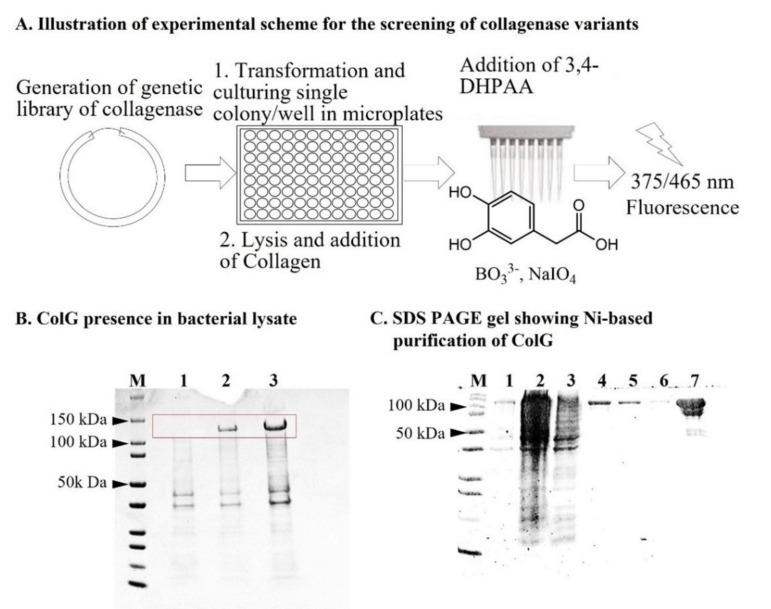
Illustration of activity assay and Coomassie-stained SDS–PAGE gels. (**A**) For screening of the genetic library of collagenase variants, single colonies were cultured in a 2 mL 96-well plate. Following lysis and separation of soluble fraction, activity was evaluated based on the 3,4-DHPAA assay. (**B**) The expression of ColG in *E. coli* BL21 cells before and following induction by IPTG. The band originated from the expression of ColG is marked by a red square. M—Marker, 1—noninduced bacterial cells, 2-bacterial cells induced with 1 mM IPTG for 4 h, 3-bacterial cells induced with 1 mM IPTG for 16 h. (**C**) Expression and purification of ColG onto a nickel column. M-Marker; 1-load; 2-sample flow through column; 4–6-wash with increased imidazole concentrations of 10, 20, and 40 mM; 7-Elution with 300 mM imidazole, [33].

Herein, we adapted a method developed for fluorescence-based detection of peptides containing an *N*-terminus Gly [33]. Based on these principles, the activity of collagenase can be monitored within a bacterial lysate following its expression and lysis. This setup enabled evaluating collagenase activity in a 96-well deep-well-plate format, and the screening of optimized protein variants from a genetic library. The repurposed assay was highly selective and enabled detection of collagenase activity with collagen in the lysates, and was thus suitable for the identification of improved variants. Figure 1A illustrates the general experimental scheme.

## 2. Results

Human collagenases have relatively high specificity and often cleave collagen at a single site. In contrast, bacterial collagenases were shown to have broad substrate specificity and are capable of digesting collagen at multiple sites. Among these bacterial enzymes, collagenases from *Clostridium histolyticum* such as collagenase G, H and T were highly characterized both structurally and biochemically [7,8,34,35]. Considering the broad range of biotechnological applications, we embarked on evaluating the activity and tested the screening of collagenase G (ColG, EC 3.4.24.3) in bacteria lysate. For this purpose, we tested the expression of ColG in *E. coli* BL21 (DE3). Following transformation of the plasmid containing the ColG gene, the bacterial culture was grown at 37 °C. At OD = 0.8, induction was executed by the addition of 1 mM IPTG. Figure 1B shows the SDS gel of the bacterial lysate before (Lane 1), 4 h after (Lane 2), and 16 h after (Lane 3) induction of protein expression by IPTG. A high level of protein expression was observed in the bacterial lysate only upon the addition of IPTG (as Lane 1 was empty), and soluble expression increased with time since induction. Therefore, this validated the inducible protein expression of ColG and indicated the potential applicability of the lysate-based screening assay. To set a benchmark for the activity of the enzyme in its purified form, we further purified the His-tagged enzyme on a nickel column. Figure 1C shows the SDS gel of the purified protein.

**Figure 2:**
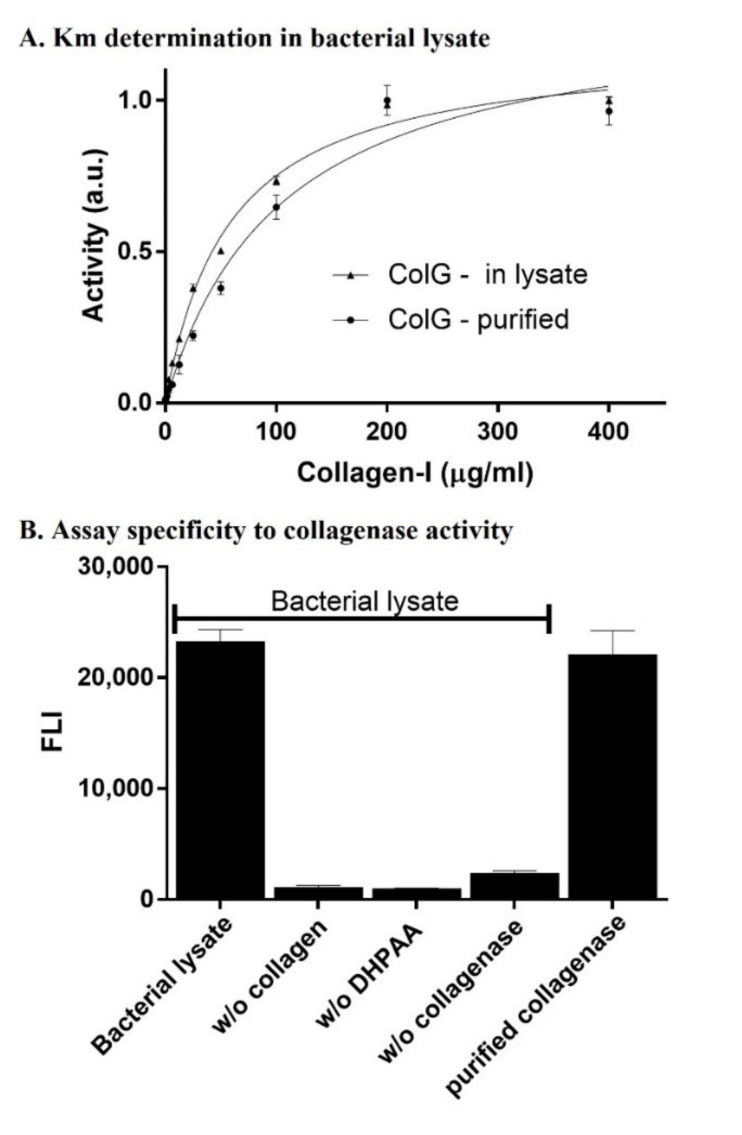
Enzymatic activity and specificity of ColG in bacterial lysate. (**A**) K_M_ determination for the purified and bacterial expressed ColG in the lysate. Triplicates of variable concentrations of the substrate collagen were incubated with each of the enzymes. Fluorescence intensity was read after the reaction reached equilibrium and normalized. (**B**) A single concentration of collagen was incubated with ColG, and assay specificity was validated via the depletion of the substrate or enzyme from the reaction. The left- and rightmost bars show the full reaction in the presence of collagen in the lysate and purified collagen, respectively. The three middle bars show the negative control reaction in which one substance of the reaction was depleted in each experiment.

After setting an established expression and purification protocol for ColG, and considering the need for an activity assay within the bacterial lysate, we tested activity using the fluorogenic reagent 3,4-dihydroxyphenylacetic acid (DHPAA) assay [33] at a range of collagen concentrations (0–400 μg/mL). A Michaelis–Menten analysis enabled the evaluating the K_M_ value of ColG towards collagen in its purified form and in the lysate (Figure 2A). In addition to verifying the activity in the lysate, this step was important for determining the optimized collagen’s concentration to be used in the screening assays. To this end, 40 µg/mL of ColG was mixed with variable concentrations of collagen in a total volume of 200 µL in each well and incubated for 1 h at 25 °C. Aliquots of 50 µL were then mixed with 50 µL of 0.75 mM 3,4-DHPAA, 50 µL of 125 mM sodium borate (pH 8.0), and 50 µL of 1.25 mM NaIO_4_ for 30 min, and incubated at 37 °C to yield the fluorescent complex of *N*-terminus Gly peptides resulting from collagen degradation by the collagenase [33]. Figure 2A shows the curves that resulted from plotting the fluorescence intensity against collagen concentration after the DHPAA reaction reached completion. The K_M_ values for the purified and lysate-based reactions were 7.1 and 23.7 µg/µL, respectively. In addition, the Vmax value of the purified enzyme was~18% higher than in the lysate, as Vmax (pure) = 1.19 × Vmax (lysate). Enzymatic reaction was performed at 25 °C as collagen type I is thermally instable above 30 °C [36].

We then investigated if the activity and readout were indeed specific to the degradation of collagen by ColG. To this end, we cultured 500 µL *E. coli* BL21 cells expressing ColG in 2 mL-deep 96-well plates. Following bacterial lysis and centrifugation, 16 µL aliquots were transferred into a new 96-well plate and incubated with 1 mg/mL collagen for 1 h at 25 °C with shaking, followed by the addition of the reaction reagents. Figure 2B shows the fluorescence intensity that resulted from the reaction at various tested conditions aimed to verify the specificity of the reaction towards collagen degradation by ColG. Significant fluorescence readouts were not observed in wells containing lysates of bacteria harboring ColG plasmid without collagen (2nd bar from left) or without DHPAA (3rd bar from left). In addition, no readout was observed in wells with lysate of *E. coli* BL21 that was not transformed with ColG plasmid (4th bar from left). On the other hand, activity of purified ColG with collagen and 3,4-DHPAA resulted in a similar intensity to that of lysates of bacteria harboring ColG plasmid. This showed that the florescence signal observed in the assay was the result of collagen’s degradation by the inducible, recombinant expressed ColG in the bacterial lysate.

Since we aimed to establish a bacterial-based assay that was amenable to screening and that could be exploited for the discovery of optimized variants of collagenases with improved rates of kinetics, identifying the optimal lysate volume and sampling time that would enable us to follow the initial velocity rate in its linear phase was important. This would ensure that optimized enzymes showed higher fluorescence intensity, which is correlated with higher activity. Thus, in our next step, we characterized the reaction kinetics in the 96-well-plate format.

**Figure 3:**
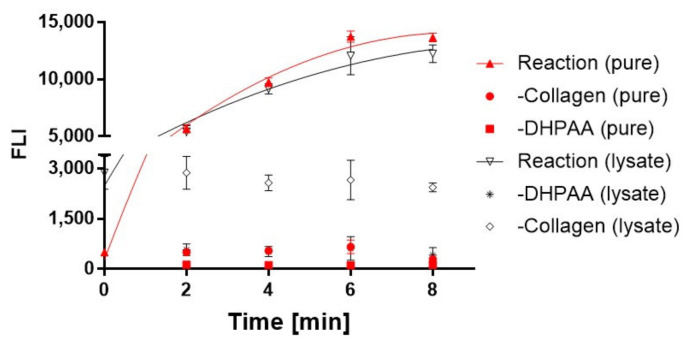
Reaction kinetics. Time-dependent evaluation of the collagen degradation by ColG. Aliquots from the reaction were analyzed based on the aforementioned assay at the designated time points. Concentration of the purified enzyme was set to 1 µg/µL, and collagen concentration was 80 µg/mL in both the purified and lysate-based assays.

Figure 3 shows several time points during the initial phase of the reaction for the purified and lysate-based enzyme. As is evident, the reaction was nearly completed after only a few minutes. This suggests that a screening assay for the discovery of optimized variants should be set such that the reaction is read during the first time points, which correspond to two to four minutes from initiation of the reaction. On the other hand, if the read will be executed towards the end of the wild-type reaction, the fluorescence intensity of an optimized variant will not show substantial difference from the reading of the wild-type, as both reactions will have reached saturation. A complementary approach could be the sampling of lower volume lysate containing the enzyme. Still, the reading of the reaction that progresses within the initial first minutes will thus enable the widest possible dynamic range of collagenase activity. Of note, relatively low nonspecific background of the reaction without collagen was observed in the lysate. This could be due to the nonspecific interaction of ColG with lysate proteins or nonspecific conversion to fluorescent signal within the course of the chemical reaction.

### 2.1. Screening, Sequencing and Modeling of a ColG Variant

Once we established a robust protocol to detect collagenase activity in the bacterial lysate as a result of inducing ColG recombinant expression, we constructed a focused genetic library of ColG. This aimed at simultaneous screening of hundreds of variants for identifying improved collagenase activity. Considering the high theoretical diversity of such a library and the currently limited capacity to screen it, the mutation rate was calibrated to incorporate about eight nonsynonymous mutations per ColG gene. The library was generated via the application of an error-prone PCR, thus resulting in random and noncontrollable mutational positions. Transformation of the library into *E. coli* BL21 cells resulted in distinct colonies that represented the genetic library, each harboring specific mutations of the variant ColG. Following transformation and plating, selected colonies from the agar plate were cultured, lysed, and tested for ColG activity in the 96-well plate.

**Figure 4:**
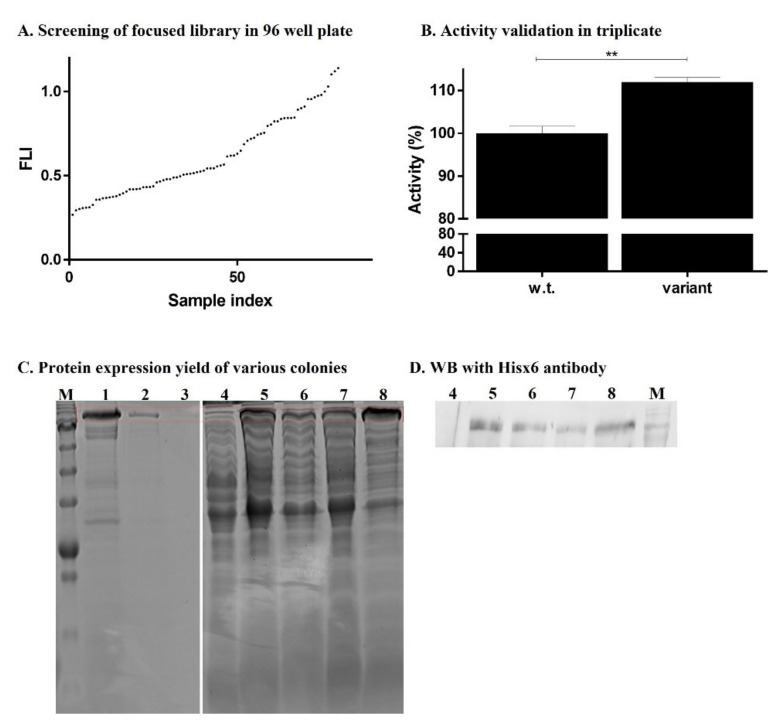
Screening of a genetic library of Col G. (**A**) The relative intensity following an enzymatic reaction of selected colonies. (**B**) The colony showing high activity was replated and colonies were grown and tested in triplicate, and activity is shown relative to the wild-type Col G. ** Calculated *p*-value was 0.0016. (**C**) SDS-PAGE gel showing ColG content in colonies cultured in a 2 mL deep-well plate for screening of ColG activity. M-Marker. Lanes 1–3–10 µg, 1 µg, and 0.1 µg of purified ColG, respectively. Lane 4-noninduced cells. Lanes 5–8-colonies expressing wild-type, phe782ser, and two randomly selected colonies, respectively. (**D**) Western blot analysis with Hisx6 antibody showing the same bacterial lysate of Lanes 4–8 as in (**C**).

Figure 4A shows screening data sorted by signal intensity relative to ColG wild-type activity. Due to the relatively large number of mutations per gene, most variants showed reduced activity compared to that of the wild-type enzyme. This was congruent with the notion that the large number of mutations hampered the structure function of the enzyme. However, few clones showed slightly increased activity, as indicated by their increased signal intensity. As a validation step, we replated the colony of the most active of these clones presenting the highest signal, grew three individual colonies of this clone, and tested each using the same screening conditions to form a triplicate. This step helped to eliminate the any false-positive signals associated with such a screening process. Figure 4B shows the intensity of the variant relative to the wild-type ColG in lysates. This indicated that the initial observation of the higher activity clone was reproducible, thus supporting the robustness of the screening assay. In the next step, we studied the molecular origin of the improved activity. Sequencing the clone revealed a unique Phe782 to Ser mutation. This residue was located at the end of the C terminus of the collagenase domain (Y119-G790); more specifically, at a polycystic kidney disease-like (PKD-like) domain [4]. Of note, differences in activity within the course of the screening may have resulted from factors such as variation in expression level, stability, and solubility of the different enzymes within each well. Still, the assay ensured that the activity was collagen degradation, and thus, although such factors cannot be quantitatively calibrated within the course of a screening, they were therefore analyzed in selected bacterial colonies, or further in the purified enzymes. Nevertheless, these traits—expression level, stability, and solubility—are highly valuable for biotechnological application. Figure 4C,D show the representative colonies that were cultured and their cellular lysate evaluated for the content of His-tagged ColG by SDS-PAGE gel and WB, respectively. It can be observed that the wild-type (Lane 5), phe782ser (ColG F782S) (Lane 6), and the additional random colony that did not show improved enzymatic activity (Lane 7) had similar expression levels. Furthermore, another colony (Lane 8) may have exhibited a higher expression level, although it did show improved activity in the enzymatic assay.

**Figure 5:**
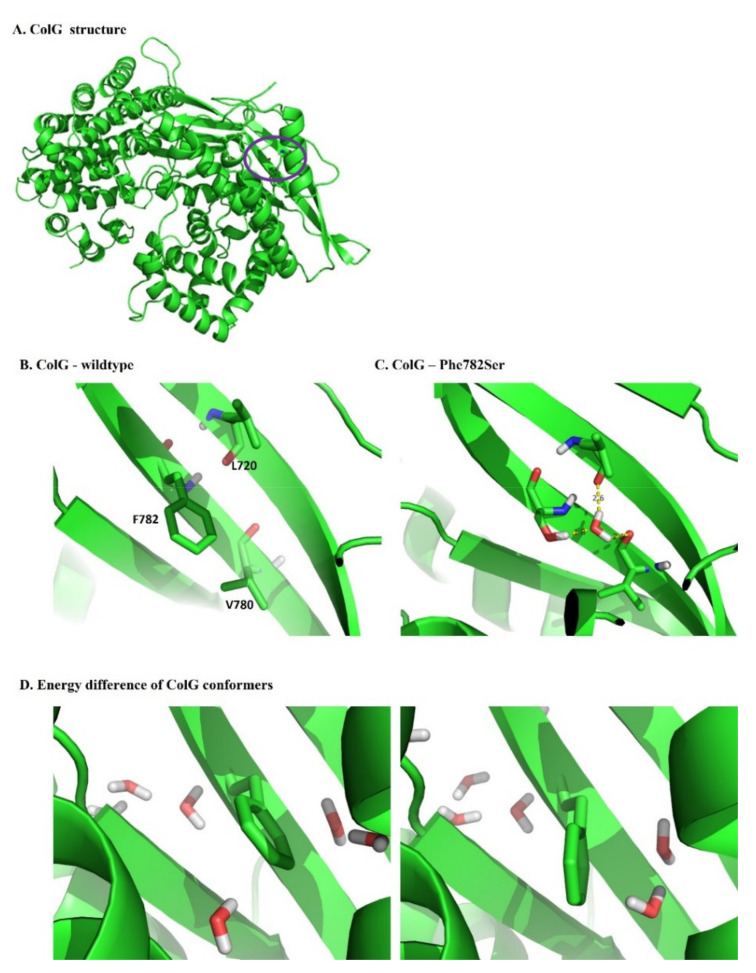
Modeling of the collagenase wild-type and ColG-F782S variant. (**A**) Structure of wild-type ColG. The position of Phe782 is circled. (**B**) Inset showing the structural architecture of Phe782. (**C**) Molecular-dynamics simulation showing the incorporation of a water molecule in the ColG-F782S variant. (**D**) Two structures calculated based on the ColG-F782S model showing unfavorable and favorable conformations of the Ser to Phe mutation.

A question may arise if structural changes appear due to the mutation. Given the assay specificity, size of the protein, and location of the mutation, we reasoned that no extreme structural change occurred, and the overall fold was maintained, primarily as activity was maintained. However, to explore a possible mechanism for the improved activity of the ColG variant, we modeled this variant following 1ns MD. Figure 5A shows the structure of the wild-type enzyme. The location of Phe782 is marked by a purple circle. Figure 5B shows an inset of the exact structural orientation of Phe782. It was positioned in a hydrophobic region, surrounded by residues such as L702, L720, L743, L747, A763, and V780, such that the hydrophilic Ser mutations seemed at first glance to destabilize the protein. However, examining the structure that resulted from molecular dynamics (Figure 5C) showed the penetration of water molecules into the cavity. This initiated the formation of a new hydrogen network, mediated by a water molecule, and connected the new Ser to Leu720 from the adjacent β sheet, as well as to Val780. The water molecule formed the basis for another layer of the H-bond network between the beta-sheets. Most importantly, the water molecule that led to the frame of the H bond layer was detected only in the MD-based frames of the variant, and did not appear in the wild-type ColG dynamic. Indeed, as Figure 5B,C indicate, it was evident that in the wild-type enzyme, the bulky F782 residue blocked such water-based interaction. Thus, despite the tight packing of this area, the mutation of the hydrophobic Phe to the hydrophilic and smaller Ser enabled penetration of the water molecule, thus contributing to the stability of the structure of the protein. To further examine the energy change, we performed a computational point mutation, from Ser back to Phe, on representative structures from the ColG-F782S dynamics. Some of these mutations showed favorable stability, mainly due to gaining back the interactions of the Phe with the hydrophobic pocket (Figure 5D, right; ∆G = −5.7 kcal/mol), while others showed unfavorable stability upon mutation, supporting our hypothesis that the H-bond network stabilized the mutant structure (Figure 5D, left; ∆G = 2.2 kcal/mol). This suggested that mutation-derived stability is based on a dynamic interplay between the hydrophobic core and the H-bond interactions.

### 2.2. Validation of Activity in the Enzyme-Purified Form

**Figure 6:**
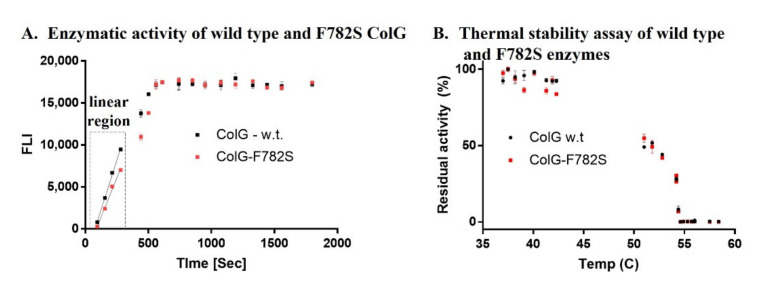
Enzymatic activity and thermal stability of purified enzymes. (**A**) Time-dependent activity of the wild-type and phe782ser variant. The inset shows the linear range of the activity. (**B**) Plot of the residual activity after incubation of the enzymes for 1 h at each temperature.

In our next step, we evaluated the collagen-degradation kinetics of the wild-type and ColG-F782S variant in their purified form. To this end, *E. coli* BL21 cells were transformed with plasmid containing the mutated gene. Induction of protein expression and subsequent purification were executed in a similar way to that of the wild-type enzyme. Figure 6A shows the time-dependent activity assay executed in a similar approach to that described in Figure 3. Purified wild-type and ColG-F782S in a concentration of 1 µg/µL were incubated with 80 µg/mL collagen. Aliquots were taken at multiple time points. It can be observed that, congruent to the results obtained in the lysate-based assay, the ColG-F782S variant exhibited slightly higher activity in its purified form. As expected, following a single round of screening, the difference in activity was not immensely high. To further explore the mechanistic basis for the improvement in activity of ColG-F782S, we evaluated the enzymes’ thermal stability. Figure 6B shows the remaining residual activity of the wild-type and variant enzymes after heating of the enzymes to various temperatures for 1 h. The enzymes were then cooled to room temperature and tested for their activity. It can be observed that there was no difference between ColG and its variant. This showed that the difference in enzymatic activity was not related to stability, but rather to activity.

## 3. Discussion

Degradation of the triple-helix collagen is an important step in the range of cellular paths, as well as in biotechnology applications. The process is most-efficiently executed by bacterial collagenases. Regardless of the degradation mechanism, a robust assay for evaluating the rate of degradation is essential for identifying inhibitors and optimized variants. To this end, and based on a previously reported assay [33], we generated a cost-effective and robust screening protocol for testing the activity of collagenases in lysate emendable in 96-well plates. The assay shown here was highly specific and sensitive, and could be executed directly on recombinant collagenase that was expressed in *E. coli* within the bacterial lysate, without the need for further purification. The latter is of immense importance for screening genetic libraries, to identify optimized variants when each colony harbors a different variant of the target enzyme. An additional important factor is the use of bacteria as the host organism. The use of more advanced systems, such as yeast or even mammalian cells, has several advantages in the field of protein-directed evolution [37]. However, *E. coli* is an attractive choice as a host organism due to its large selection of cloning vectors and strains, as well as its rapid and well-regulated growth rate. A major disadvantage of the proposed strategy is its relatively low throughput compared to other display-based methods. However, computational predictions and the semirational design of the mutational space can compensate the screening space by generating a focused library based on in silico filtering, thus enabling an efficient screening strategy. The current work employed the screening of 96-well plates, in which few colonies showed slight improved activity, but the activity of most of the samples was reduced. It is likely that the exact number of mutations per gene should be calibrated for each collagenase enzyme, and that the insertion of eight mutations per gene was too high. Indeed, the selected colony had a single mutation in the amino acid level. Thus, thorough calibration of the mutational number should be considered.

An additional factor to note is the molecular-level origin of the improved activity. Given the relatively large molecular weight of the target protein, mutations could arise in regions not directly related to the enzymatic activity. However, such mutations may lead to enhanced stability or higher expression levels of the variant. In the current protocol, all the mutations translated to a higher fluorescent readout. Thus, the structure-function aspects must be further deciphered in order to integrate successful substitutions into a potent protein variant. 

## 4. Materials and Methods

### 4.1. ColG Expression and Purification

The plasmid expressing ColG was a gift from Hans Brandstetter [8]. The gene encodes residues Tyr119-Lys1118 with an *N*-terminus 6xHIS tag, followed by a cleavable TEV site. The plasmid was transformed to *E. coli* BL21 and cells; at OD = 0.8, protein expression was induced by the addition of 1 mM IPTG. Following overnight culturing at 25 °C, the cells were harvested. The harvested cells were resuspended in 40 mL of 50 mM Tris-HCl (pH 8.0), 300 mM NaCl, and 30 mM imidazole. The suspended cells were then disrupted by sonication, and the insoluble fraction was removed by centrifugation for 20 min at 15,000× *g*. The supernatant was applied to a 5 mL column of nickel beads. After washing the resin, the protein was eluted with the addition of 300 mM imidazole. For the execution of the enzymatic assay in the 96-well format, bacterial cells were disrupted by the addition of ‘Bug-Buster’ reagent according to the manufacturer’s instructions, with the addition of 0.4 mg/mL of lysozyme and 250 units/10 mL benzonase.

### 4.2. Enzymatic Assay

An enzymatic assay was repurposed based on a previously described protocol [33]. Purified enzyme or bacterial lysate was incubated with collagen-I, and aliquots were mixed with 50 mM Tris buffer (pH 7.5), 5 mM CaCl2, and DDW, in a total volume of 200 µL in each well, for 2–4 min incubation at 37 °C. Aliquots of 50 µL were then mixed with 50 µL of 0.75 mM 3,4-DHPAA, 50 µL of 125 mM sodium borate (pH 8.0), and 50 µL of 1.25 mM NaIO4, then incubated for 30 min at 37 °C. The fluorescence intensity of the reaction mixture was measured with a spectrofluorometer. The excitation and emission maxima were 375 nm and 465 nm, respectively.

### 4.3. Construction of a Genetic Library

A genetic library was constructed from the ColG gene with the GeneMorph II Random Mutagenesis Kit (Agilent, Santa Clara, CA, USA), adjusted to produce an average of 6 nonsynonymous mutations per gene. Following mutagenesis PCR, libraries were cloned back into the original vector. The cloned vectors were transformed into *E. coli* BL21 cells and plated on an LB plate supplemented with 100 mg/mL ampicillin. Individual colonies were randomly selected and grown overnight in 96-well deep-well plates containing 500 mL LB supplemented with 100 mg/mL ampicillin at 37 °C, with shaking. The overnight culture was used to inoculate (at 1:20 dilution) fresh 500 mL LB supplemented with 200 mg/mL ampicillin in 96-well deep-well plates. The cells were grown at 30 °C, with shaking, for about 4 h, to an OD600 = 0.8, and induced with 1 mM IPTG to induce expression of the ColG variants. Following overnight incubation at 25 °C, the cells were pelleted and resuspended in Bug-Buster lysis buffer for further enzymatic activity assay.

### 4.4. Modeling and MD Simulation

All simulations were performed using the GROMACS software version 2020.1 [38,39] and the Amber force field [40,41]. The coordinates of the protein were based on a unit of collagenase G from Clostridium histolyticum pdb# 4ARE [8]. In this structure, the calcium ion is missing, and is replaced by a water molecule. We used the homologue collagenase H pdb# 4ARF to align and insert the calcium ion, together with its two water ligands, to the feasible position in the protein. The structure was first prepared for simulation using the maestro module of Schrodinger [42]. This comprised filling missing side chains, adding hydrogens in correct ionization states, short optimization of the hydrogens, and finally a short minimization to relax strained bond angles and clashes. The protein was embedded in a cubic water box, with 1.2 nm of solvent on all sides of the protein. Water molecules were described using the TIP3P model. Counter ions (Cl^−^ and Na^+^) were inserted to achieve a neutral simulation cell. Energy minimization was carried out using the steepest descent algorithm, followed by the conjugate gradient algorithm. The system was then equilibrated with a 100 ps MD simulation in the canonical (NVT) ensemble, using the modified Berendsen thermostat [43] for fixing the temperature of the system at 310. This was followed by a 100 ps MD simulation in the isothermal–isobaric (NPT) ensemble, using the Parrinello–Rahman pressure-coupling method for maintaining the pressure at a fixed 1 bar. Position restraints on the bonds to hydrogen atoms were applied using the Lincs algorithm [44]. These were in addition to distance restraints on the Ca-water ligands bonds in the wild-type only. The following MD simulation, starting from the previous NPT simulation, was equilibrated for another 1 ns with no position restraints. All images, as well as alignments of structures and the initial structure for the F782S mutation, were maintained using Pymol software [45].

### 4.5. Chemicals

The collagen-I at a concentration of 1 mg/mL (Cat#92695), 3,4-DHPAA, and boric acid were purchased from Merck (Sigma), Darmstadt, Germany. The NaIO_4_ was purchased from Thermo Fisher Scientific, Waltham, MA, USA. The Bug-Buster used for bacterial lysis was purchased from Merck (Millipore). The lysozyme was purchased from Merck, Darmstadt, Germany, and the benzonase was purchased from Santa Cruz, Dallas, TX, USA. All other chemicals and reagents were purchased from Merck.

## Data Availability

The data presented in this study are available on request from the corresponding author.
